# The human anti-IL-1β monoclonal antibody ACZ885 is effective in joint inflammation models in mice and in a proof-of-concept study in patients with rheumatoid arthritis

**DOI:** 10.1186/ar2438

**Published:** 2008-06-05

**Authors:** Rieke Alten, Hermann Gram, Leo A Joosten, Wim B van den Berg, Joachim Sieper, Siegfrid Wassenberg, Gerd Burmester, Piet van Riel, Maria Diaz-Lorente, Gerardus JM Bruin, Thasia G Woodworth, Christiane Rordorf, Yannik Batard, Andrew M Wright, Thomas Jung

**Affiliations:** 1 Department of Internal Medicine II, Rheumatology, Schlosspark-Klinik Teaching Hospital Charité University Medicine Berlin, Heubnerweg, D-14059 Berlin, Germany; 2 Novartis Institutes of Biomedical Research, Basel, CH-4002 Basel, Switzerland; 3 Rheumatology Research and Advanced Therapeutics, Radboud University Nijmegen Medical Centre, Nijmegen 6500 HB, The Netherlands; 4 Department of Rheumatology, Klinikum Benjamin Franklin, Hindenburgdamm, D-12200 Berlin, Germany; 5 Department of Rheumatology, Evangelisches Fachkrankenhaus, Rosenstraße, D-40882 Ratingen, Germany; 6 Department of Rheumatology, Charité, Charitéplatz, D-10117 Berlin, Germany; 7 Department of Rheumatology, University Medical Center St Radboud, Nijmegen 6500 HB, The Netherlands; 8 Novartis Exploratory Development, Werk St. Johann, Forum 1, CH-4002 Basel, Switzerland

## Abstract

**Introduction:**

IL-1β is a proinflammatory cytokine driving joint inflammation as well as systemic signs of inflammation, such as fever and acute phase protein production.

**Methods:**

ACZ885, a fully human monoclonal antibody that neutralizes the bioactivity of human IL-1β, was generated to study the potent and long-lasting neutralization of IL-1β in mechanistic animal models as well as in a proof-of-concept study in patients with rheumatoid arthritis (RA).

**Results:**

The mouse IL-1 receptor cross-reacts with human IL-1β, and it was demonstrated that ACZ885 can completely suppress IL-1β-mediated joint inflammation and cartilage destruction in mice. This observation prompted us to study the safety, tolerability and pharmacodynamic activity of ACZ885 in RA patients in a small proof-of-concept study – the first to be conducted in humans. Patients with active RA despite treatment with stable doses of methotrexate were enrolled in this dose escalation study. The first 32 patients were split into four cohorts of eight patients each (six were randomly assigned to active treatment and two to placebo). ACZ885 doses were 0.3, 1, 3 and 10 mg/kg, administered intravenously on days 1 and 15. To explore efficacy within 6 weeks of treatment, an additional 21 patients were randomly assigned to the 10 mg/kg cohort, resulting in a total of 20 patients dosed with 10 mg/kg and 15 patients treated with placebo. There was clinical improvement (American College of Rheumatology 20% improvement criteria) at week 6 in the 10 mg/kg treatment group; however, this did not reach statistical significance (*P *= 0.085). A statistically significant reduction in disease activity score was observed after 4 weeks in the 10 mg/kg group. Onset of action was rapid, because most responders exhibited improvement in their symptoms within the first 3 weeks. C-reactive protein levels decreased in patients treated with ACZ885 within 1 week. ACZ885 was well tolerated. Three patients receiving ACZ885 developed infectious episodes that required treatment. No anti-ACZ885 antibodies were detected during the study.

**Conclusion:**

ACZ885 administration to methotrexate-refractory patients resulted in clinical improvement in a subset of patients. Additional studies to characterize efficacy in RA and to determine the optimal dose regimen appear warranted.

**Trial Registration:**

ClinicalTrials.gov identifier NCT00619905.

## Introduction

Rheumatoid arthritis (RA) is a chronic inflammatory disease of the joints that is associated with destruction of cartilage and bone. In addition to its local destructive nature, it also can have a pronounced systemic inflammatory component, presenting as fever, headache and fatigue, and may even affect other organs such as the skin, liver, spleen and lymph nodes. The contribution made by proinflammatory cytokines such as tumour necrosis factor (TNF)-α and IL-1 has been validated in preclinical animal models and in humans [1,2].

Current therapy targeting IL-1 in RA is based on a recombinant form of the IL-1 receptor antagonist (IL-1Ra; anakinra), which binds to the IL-1 receptor without eliciting a signal and therefore competes with the binding of the two natural ligands, namely IL-1α and IL-1β. As compared with TNF-α inhibitors, it appears that treatment with the recombinant IL-1Ra is less efficacious in RA [3]. However, it is unclear whether this is due to biological superiority of TNF-α over IL-1 in RA pathogenesis [4] or whether the approach to block the IL-1 receptor with anakinra is only partly effective in neutralizing IL-1 activity *in vivo *because of short half-life or inability to achieve sufficient exposure at the target site with the current dosing regimen [5].

The main elicitor of inflammatory responses in preclinical animal models appears to be IL-1β and not IL-1α. In murine collagen-induced arthritis [6] and antigen-induced arthritis [7,8], inflammation and cartilage degradation were inhibited by blocking IL-1β but not IL-1α. In RA patients, IL-1β is over-expressed in inflamed synovial tissue, in particular in the lining layer and in sublining cells [9], and it is elevated in draining lymph from affected joints [10]. Furthermore, IL-1β is localized to the synovial pannus in close proximity to bone and cartilage [11], and cartilage from arthritis patients exhibits upregulation of IL-1β mRNA as compared with normal cartilage. Increased levels of IL-1β in synovial fluid [12] have been described and were found to correlate with histological features of RA [13,14]. Treatment with anakinra demonstrated improvement in histological characteristics of RA joint pathology [15], and evidence was provided that it delays the progression of radiographic joint damage [16]. Thus, there is reason to believe that IL-1β is a crucial cytokine that drives inflammation and joint destruction in RA.

We report here results of preclinical studies and a proof-of-concept study in patients with RA, using a fully monoclonal antibody (ACZ885) directed against human IL-1β. ACZ885 is expected to have the half-life of a typical IgG_1 _antibody, thus ensuring full neutralization of IL-1β over a longer period of time as compared with recombinant IL-1Ra; the therefore study explored pharmacodynamic effects in RA patients (secondary to an evaluation of safety).

## Materials and methods

### *In vivo *activity of ACZ885 in a human IL-1β-dependent mouse model of joint inflammation

Cells of the murine fibroblast line 3T3NIH were transfected with retroviral vector DFG-hIL-1β-neo, which contains the cDNAs for human (h)IL-1β and neomycin phosphotransferase (neo^R^), as described previously [17]. The 3T3NIH fibroblast cell line was derived from a DBA background. One clone derived from the transduction, namely IB2 (3T3-hIL-1β), was selected, which produced high levels of hIL-1β (220 ng hIL-1β/24 hours per 10^6 ^cells) *in vitro*. hIL-1β-induced arthritis was induced by intra-articular injection of 10^4 ^IB2 cells into the right knee joints of DBA-1 mice. Mice were injected with ACZ885 or isotype control antibody CHI621, which is specific for human CD25 and does not cross-react with mouse cells or tissue. All mice were housed in filter top cages, and water and food were supplied *ad libitum*. The mice were studied at age 10 to 14 weeks. All animal experiments were approved by the Nijmegen University Animal Ethics Committee.

Administration of antibodies was intraperitoneal 2 hours before intra-articular injection of the IB2 clone. Joint inflammation was quantified using the ^99m^Tc uptake method [18]. By external gamma counting, this method measures the accumulation of a small radioisotope at the site of inflammation caused by locally increased blood flow and tissue swelling. The severity of inflammation is expressed as the ratio of ^99m^Tc uptake in the right (inflamed) to that in the left (control) knee joint.

Proteoglycan synthesis was assayed as follows. Patellae with minimal surrounding tissue were placed in RPMI 1640 medium with glutamax, gentamycin (50 μg/ml) and ^35^S-sulphate (0.74 MBq/ml). After 3 hours of incubation at 37°C in a carbon dioxide incubator, patellae were washed in saline three times, fixed in 4% formaldehyde and subsequently decalcified in 5% formic acid for 4 hours. Patellae were punched out of the adjacent tissue, dissolved in 0.5 ml Luma Solve at 65°C (Omnilabo, Breda, The Netherlands), and after addition of 10 ml Lipoluma (Omnilabo) the ^35^S content was measured using liquid scintilation counting. For histological analysis, whole knee joints were removed and fixed in 4% formaldehyde for 7 days before decalcification in 5% formic acid and processing for paraffin embedding. Tissue sections (7 μm) were stained with haematoxylin/eosin or safranin O/fast green. Histopathological changes in the knee joints were scored in the patella/femur region on five semi-serial sections, spaced 140 μm apart. Scoring was performed on decoded slides by two separate observers, using the following parameters. In the haematoxylin/eosin stained slides the amount of cells infiltrating the synovial lining and the joint cavity was scored from 0 to 3. Proteoglycan depletion was scored in the safranin O stained slides on a scale from 0 to 3 (ranging from stained cartilage to fully destained cartilage). Cartilage damage was scored from 0 to 3 (ranging from unaffected cartilage to maximum chondrocyte death and cartilage destruction).

### Design of clinical study

This was a randomized, double-blind, placebo-controlled, dose escalation study conducted to explore the safety, tolerability, pharmacokinetics and pharmacodynamics of ACZ885 in patients with active RA, despite ongoing treatment with a stable dose of methotrexate (MTX) of 15 mg/week or more for at least 3 months (CACZ885A2101). It is the first of its kind to be conducted in humans. Patients receiving stable doses of 10 mg/week methotrexate could be included if the patients could not tolerate higher doses.

Included patients were required to have a diagnosis of RA (American College of Rheumatology [ACR] 1987 revised classification for criteria for RA) with a disease duration of at least 6 months before randomization; active disease at screening; baseline evaluation (same evaluator) indicating more than six tender and six swollen joints out of 28 examined (including any effused joint); and either Westergren erythrocyte sedimentation rate of 28 mm/hour or greater, or C-reactive protein (CRP) of at least 6 mg/l. The inclusion criteria also required patients were have failed at least one disease-modifying antirheumatic drug in the past, but not deemed 'refractory to all therapies'. Their current treatment regimen included at least 15 mg methotrexate/week and with the current dose stable for approximately 3 months. Patients were required to have an otherwise stable RA therapeutic regimen, consisting of either a stable dose of nonsteroidal anti-inflammatory drugs and/or a stable dose of oral corticosteroids (prednisone or equivalent < 10 mg/day) for at least 4 weeks before randomization.

This multicentre study was conducted in accordance with GCP (Good Clinical Practice) guidelines and the Helsinki Declaration in three countries in Europe. The study protocol and patient informed consent form was approved by local ethics committees and health authorities. The primary objective was to evaluate the safety and tolerability of ACZ885 administered as an intravenous infusion. Secondary objectives included assessment of the preliminary biologic activity/pharmacodynamics of ACZ885 using ACR criteria for clinical response and Disease Activity Score using 28 joint counts (DAS28), evaluation of the pharmacokinetics of ACZ885 and examination of the potential immunogenicity of ACZ885.

**Figure 1:**
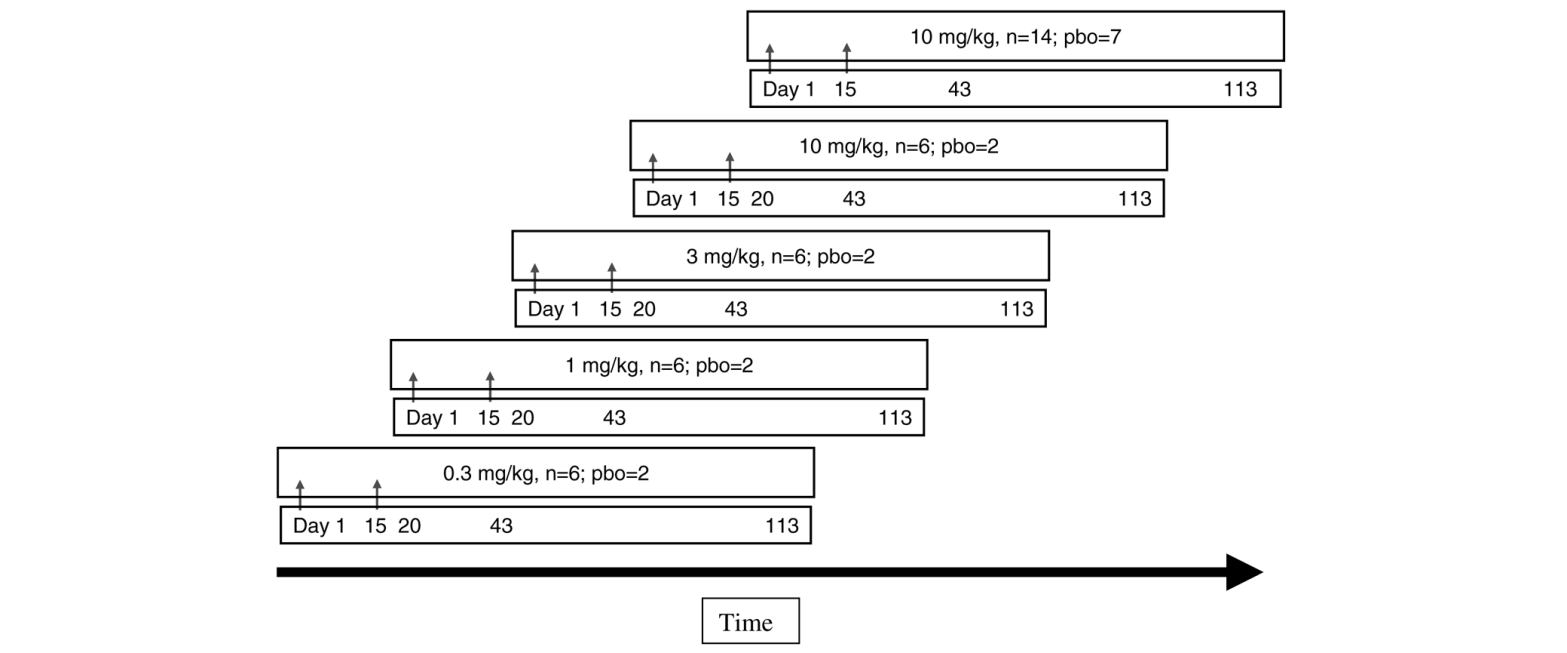
Study design. Sequential dose-escalation, randomized, double-blind, placebo controlled study design with an extension at the highest dose level. ACZ885 was administered intravenously on days 1 and 15 (arrows). Safety data generated up to day 20 in each cohort were reviewed before escalating to the next higher dose level. Independent observer efficacy assessments were made at day 1, before dosing, and day 43, in addition to weekly investigator assessments up to Day 43. The end of study was on day 113. After safety review of the first six patients at 10 mg/kg, an extension cohort was started, which included 14 patients on ACZ885 and seven patients on placebo (pbo).

The initial dose escalation phase of the study consisted of four cohorts (Figure 1). In each cohort six patients were randomly assigned to receive ACZ885 and two patients were randomly assigned to receive placebo. In the first cohort, patients randomly assigned to ACZ885 received 0.3 mg/kg intravenously on days 1 and 15. Safety data from a minimum of six out of eight patients in this cohort up to day 20 were reviewed prior to proceeding with the study and starting the next higher dose. Patients randomly assigned to ACZ885 in the second cohort received 1 mg/kg, in the third cohort 3 mg/kg, and finally in the fourth cohort 10 mg/kg. Safety data from the 10 mg/kg cohort were found to raise no concern that might prevent proceeding with the study, so this cohort was extended to increase the sample size, such that in total 20 patients received 10 mg/kg intravenous on days 1 and 15, and a total of 15 patients received placebo. Thus, a total of 53 patients with RA were enrolled.

ACR response criteria were evaluated at baseline and on days 8, 15, 22, 29 and 43 by the investigator, and at baseline and day 43 by a blinded observer at each site who was not otherwise involved in the study. Overall, there was no evidence of a systematic bias between the blinded observer and the investigator assessments. Patients were further followed for safety and pharmacokinetic assessments, and a formal end of study was reached at day 113. Investigators were free to change treatment after day 43 as they deemed necessary.

### Statistical analyses

Statistical analyses were performed on the ACR 20%, 50% and 70% response criteria (ACR20, ACR50 and ACR70) rates. Separate statistical analyses were performed on the response rate at day 43 (assessed by a blinded observer) and the response rate up to and including day 43. Fisher's exact test was used to compare treatment groups. From these analyses, one sided *P *values are presented. The primary comparison was the comparison between the 10 mg/kg treatment group and placebo at day 43. For the DAS28 measurements, a repeated measures mixed effect analysis was performed. Baseline DAS28 was included in the model as a covariate. An unstructured correlation structure was assumed to adjust for correlation within patients. From this model the adjusted mean difference for each treatment group versus placebo at each time point was calculated, along within its 95% confidence interval. No multiplicity adjustments were made to the confidence intervals. A similar analysis was performed on the CRP measurements; however, because of the skewness of the CRP measurements, the analysis was performed on the log_e _scale and results were back-transformed onto the original scale.

The inclusion of six patients per dose group in each escalation step provided an 80% probability that at least one patient would report an adverse event that has an underlying incidence rate of 24%. With 20 patients receiving the highest ACZ885 dose (six patients in the last escalation step plus 14 patients in the expansion phase) and 15 patients receiving placebo (two patients in each of the four escalation steps plus seven patients in the expansion phase), there was at least 80% power to detect a statistically significant difference in ACR20 response rate comparing ACZ885 with placebo, assuming an underlying odds ratio of 6, a 5% significance level and a one-side test approach. Given an anticipated placebo ACR20 response rate of 20%, an odds ratio of 6 would translate into an ACR20 response rate of 60% for the ACZ885 treatment group.

## Results

### ACZ885 is a human antibody that binds to human IL-1β with high affinity and neutralizes human IL-1β *in vitro *and *in vivo*

ACZ885 was derived by subjecting genetically engineered mice carrying part of the human immunoglobulin repertoire to immunization with human IL-1β. The antibody was initially identified from a hybridoma derived from these mice because of its ability to bind human IL-1β with high affinity (about 40 pmol/l). No cross-reactivity toward rodent IL-1β or human IL-1α was observed for ACZ885. The antibody was found to neutralize the bioactivity of human IL-1β on primary human fibroblasts *in vitro *with an 50% inhibitory concentration of 44.6 pmol/l (7.1 ± 0.56 ng/ml; *n* = 6).

**Figure 2:**
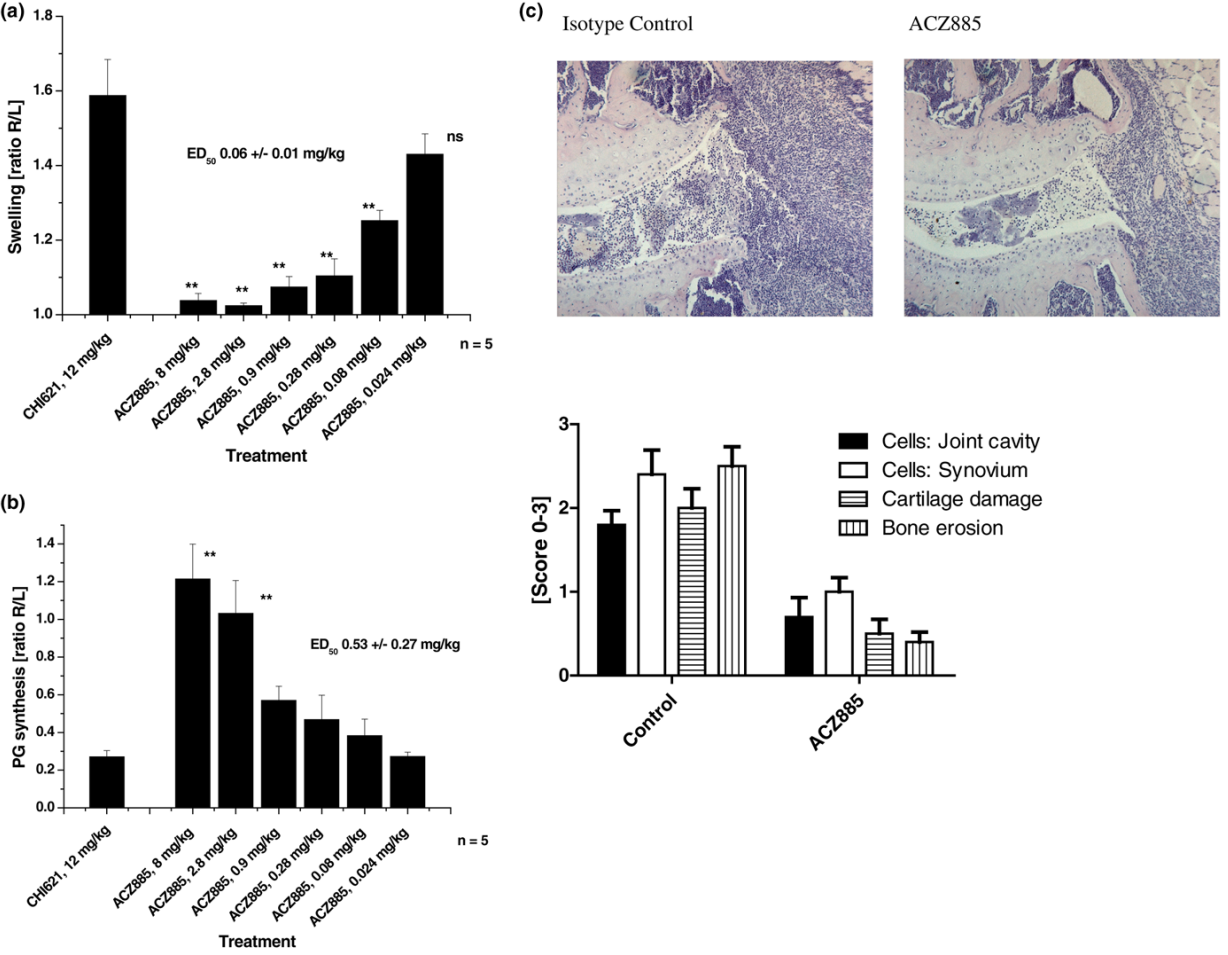
Blocking of IL-1 in mouse models of arthritis. **(a) **Inhibition of swelling. Mice were given different doses of ACZ885 or a control anti-CD25 antibody (CHI621, 12 mg/kg) intraperitoneally before injection of 10,000 3T3-huIL-1β cells into the right hind knee joint. Swelling was measured 3 days after cell injection (as described in Materials and methods) and is expressed as the ratio between the right (treated) and left (untreated) joint. The results presented represent the mean ± standard error or the mean (SEM; *n* = 5). **P < 0.01 by analysis of variance followed by Dunnett's test for multiple comparisons *post hoc*. NS, not significant. **(b) **Proteoglycan (PG) synthesis by chondrocytes in explanted patellae was measured by incorporation of ^35^S labelled sulphate in isolated cartilage from treated (right) and untreated (left) knee joints. The results are given as the ratio of ^35^S incorporation between right and left knee cartilage and represent the mean ± SEM (*n* = 5). Statistical analysis of the treated groups versus the control group (CHI621) was performed by analysis of variance followed by Dunnett's test for multiple comparisons *post hoc*. *P < 0.05; **P < 0.01. ED_50_, dose needed to achieve a mean of 50% effect. **(c) **Histology was analyzed at day 3 after local injection of 3T3-hIL-1β producing cells. Section of a mouse knee joint treated with isotype control antibody or AC885 are shown. Hematoxylin and eosin staining, original magnification (200×). Quantitative evaluation of the slides was done as described in Materials and methods and is presented in the graph. *n* = 5 joints per group; all comparisons between active and control were significant (*P *< 0.05, Mann-Whitney U-test).

IL-1β requires highly potent antagonists in large excess to neutralize its biological activity *in vivo*. Anti-cytokine antibodies with low neutralizing capacity can potentially lead to enhanced bioactivity of the targeted cytokine by prolonging its half-life *in vivo *[19]. In light of this potential complication, we considered demonstration that ACZ885 is devoid of any biologically relevant carrier effect in an *in vivo *model to be an important prerequisite for conducting clinical trials. Therefore, we created an artificial mouse model of joint inflammation that relies upon continuous secretion of human IL-1β by transfected mouse NIH3T3 cells injected into one knee joint. Whereas IL-1β driven inflammation becomes apparent after 24 hours and leads to total destruction of the joint within 7 to 10 days, controlateral joints or joints receiving control 3T3 cells do not exhibit signs of inflammation. Application of ACZ885 intraperitoneally 2 hours before injecting the IL-1β producing cells completely suppressed joint swelling, with a 50% effective dose (the dose effective to reduce inflammation by a mean of 50%; ED_50_) of 0.06 mg/kg (Figure 2a). Also, treatment with ACZ885 restored the ability of cartilage explants to synthesize proteoglycan, which is otherwise suppressed in IL-1β exposed cartilage (Figure 2b). In addition, histopathology taken at day 3 after injection of 3T3-hIL-1β cells revealed that administration of AC885 protected against severe joint destruction (Figure 2c). Apart from the strong reduction of the joint inflammation (numbers of cells in the synovial tissues), no bone erosion was noted in the AC885 treated animals compared with controls. Thus, ACZ885 has sufficient potency to neutralize fully the biological activity of human IL-1β, even when it is produced at high concentrations *in vivo*, and it does not significantly prolong IL-1 activity.

### Proof-of-concept clinical trial in patients with RA

A proof-of-concept study to explore safety and tolerability, pharmacokinetics and pharmacodynamics was conducted in patients with established RA with ongoing disease despite stable treatment with MTX. (Some of the data from this study were presented at EULAR [European League Against Rheumatism] in 2006 [20].) An overview of patient demographics at baseline is given in Table 1, and the design of the study is presented in Figure 1.

**Table 1:** Baseline demographics of participating patients

Characteristic	ACZ885				
	
	0.3 mg/kg (*n* = 6)	1.0 mg/kg (*n* = 6)	3.0 mg/kg (*n* = 6)	mg/kg (*n* = 20)	Placebo (*n* = 15)
Age (years; mean ± SD)	61 ± 7	53 ± 14	57 ± 7	50 ± 11	55 ± 10
hsCRP (mg/l; median [range])	17.8 (1.6–95.3)	43.8 (5.8–89.3)	3.0 (2.0–43.4)	11.1 (1.5–28.4)	6.4 (0.2–66.2)
DAS28 (mean ± SD)	6.5 ± 0.8	6.6 ± 1.2	6.7 ± 1.5	6.2 ± 0.7	6.7 ± 0.6
Number of tender joints (mean ± SD)	17 ± 9.2	15 ± 6.2	22 ± 8.5	17 ± 7.3	20 ± 6.3
Number of swollen joints (mean ± SD)	13 ± 6.3	12 ± 4.3	12 ± 7.4	11 ± 4.8	12 ± 5.2
Methotrexate dose (*n*/mg median [range])^a^					
Oral	6/17.5 (15–25)	5/15 (10–15)	4/15 (15–15)	13/15 (10–22.5)	5/15 (10–20)
Parenteral	0	1/10 (10–10)	2/22.5 (20–25)	7/15 (10–22.5)	10/15 (10–25)

^a^Numbers of patients receiving 15 mg/week were 2, 4, 4, 10 and 6 in the 0.3, 1, 3, 10 mg/kg and placebo cohorts, respectively. SD, standard deviation.

In the 10 mg/kg cohort there were six out of 19 (32%) ACR20 responders at day 43, as assessed by the blinded observer, versus one out of 15 (7%) in the placebo group (one-sided *P *= 0.085; Table 2). ACR50 and ACR70 improvements were observed at the highest dose levels (3 and 10 mg/kg) in a total of four out of 25 patients, but no such responses were observed in the placebo group. Using the investigator assessments, 10 out of 20 patients (50%) achieved an ACR20 and four out of 20 an ACR50 or better response at any time point up to and including day 43 (Table 2). There was a statistically significant difference compared with placebo in terms of ACR20 improvement when all ACZ885 cohorts (one-sided *P *= 0.043) or when the 3 mg/kg and 10 mg/kg cohorts were pooled (one-sided *P *= 0.035). Nine out of the 10 ACR20 responders satisfied criteria for response within 3 weeks after the first infusion. The time to achieve an ACR50 response was 1 week in one patient, 3 weeks in a second patient, and 4 weeks in two patients. Under placebo, three out of 15 patients exhibited an observable ACR20 response during the 6-week period of observation. One patient had an ACR20 response after 1 week, which disappeared thereafter. A second patient had an ACR20 response at weeks 1 and 3 but not on any other occasion. The third patient had an ACR20 response after 3 weeks which continued after day 43.

**Table 2:** Clinical improvement as assessed by ACR criteria

Assessor (time point)	Response	ACZ885				Placebo (*n* = 15)	*P *value (10 mg/kg versus placebo)
		
		0.3 mg/kg (*n* = 6)	1.0 mg/kg (*n* = 6)	3.0 mg/kg (*n* = 6)	10 mg/kg (*n* = 19/20^a^)		
Blinded observer (day 43)	ACR20	1 (17%)	0 (0%)	4 (67%)	6 (32%)	1 (7%)	0.085
	ACR50	0 (0%)	0 (0%)	1 (17%)	3 (16%)	0 (0%)	0.162
	ACR70	0 (0%)	0 (0%)	0 (0%)	2 (11%)	0 (0%)	0.305
Investigator (any time within 6 weeks of treatment start)	ACR20	3 (50%)	2 (33%)	4 (67%)	10 (50%)	3 (20%)	0.070
	ACR50	0 (0%)	0 (0%)	2 (33%)	4 (20%)	0 (0%)	0.093
	ACR70	0 (0%)	0 (0%)	0 (0%)	3 (15%)	0 (0%)	0.174

Shown are the number and percentage of patients achieving 20%, 50%, or 70% improvement in terms of American College of Rheumatology (ACR) criteria (ACR20, ACR50 and ACR70, respectively) at day 43 as assessed by a blinded observer and at any time point within 6 weeks of treatment start as assessed by the investigator. ^a^Note that for one patient in the 10 mg/kg treatment group, the ACR criteria assessment by a blinded observer was not conducted at day 43.

**Figure 3:**
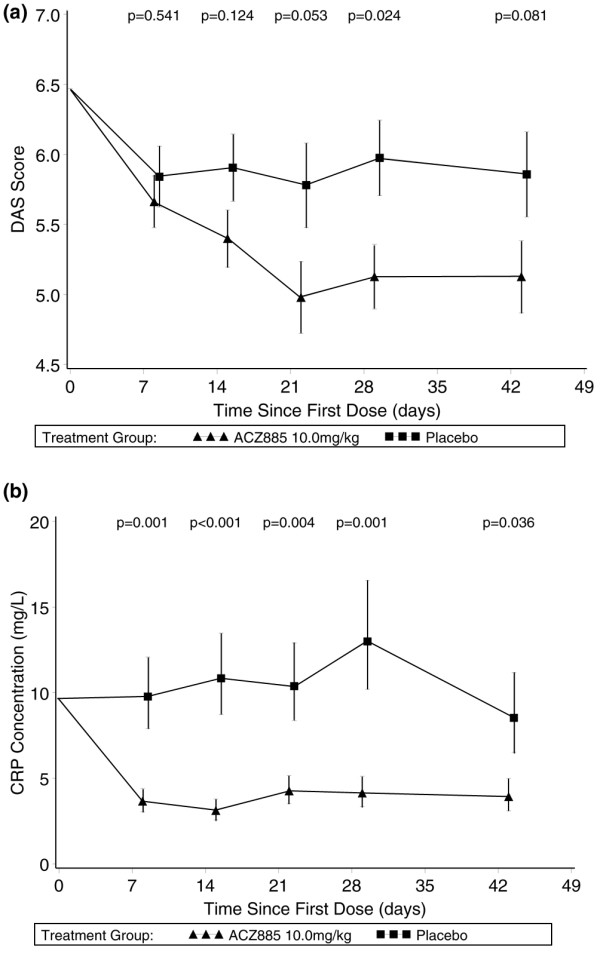
Reduction in disease activity and CRP levels following ACZ885 therapy in RA patients. **(a) **Mean disease activity (Disease Activity Score using 28 joint counts [DAS28]) levels over time. Baseline adjusted mean and standard error of the mean (SEM) from the repeated measures analysis are presented for patients treated with ACZ885 10 mg/kg (*n* = 20) and placebo (*n* = 15). *P *values for comparison of ACZ885 10 mg/kg versus placebo at each time point are presented. **(b) **Geometric mean C-reactive protein (CRP) levels over time. Baseline adjusted mean and SEM from the repeated measures analysis are presented after transformation back onto the original scale for patients treated with ACZ885 10 mg/kg (*n* = 20) and placebo (*n* = 15). *P *values for comparison of ACZ885 10 mg/kg versus placebo at each time point are presented. Normal range of CRP was 0 to 8.4 mg/l.

Figure 3a shows the mean reduction in DAS28 in the cohort treated with 10 mg/kg as compared with placebo. The *P *values for the treatment comparisons at days 29 and 43 were 0.024 and 0.081, respectively. Using the EULAR response criteria, a subset of five out of 20 patients treated with 10 mg/kg ACZ885 achieved a good response, and 10 out of 20 achieved a moderate response at any time point up to and including day 43 under 10 mg/kg. Four out of five patients who were classified as good responders achieved the necessary DAS28 reduction within the first 3 weeks of treatment, indicating a rapid onset of action.

The fact that about 50% of all patients exhibited clinical benefit within the first 6 weeks of treatment prompted us to analyze CRP levels under therapy as a potential marker of response versus nonresponse. As shown in Figure 3b, there was a substantial mean reduction in and normalization of CRP within a few days after commencing treatment with 10 mg/kg ACZ885. Similarly, CRP was also reduced upon treatment in the other active treatment cohorts (data not shown). As demonstrated in Table 3 for the 10 mg/kg cohort, a reduction in CRP at day 43 was required to achieve a ACR20 or higher response; however, eight subjects had a more than 50% reduction in CRP but had no detectable clinical response based on ACR20 criteria. Thus, CRP levels declined under ACZ885 treatment in the majority of patients (14/18 patients amenable for analysis), but only six of the CRP responders achieved an ACR20 response at day 43. Within the placebo cohort (Table 3), the majority of patients (*n* = 10) did not exhibit any meaningful CRP reduction (<20%); however, CRP was reduced by more than 50% in three patients, but this was not associated with an ACR20 response.

**Table 3:** Relationship between CRP reduction and ACR response following ACZ885 treatment

Group	CRP reduction at day 43 versus baseline	ACR response at day 43		
		
		<20%	20% to <50%	50% or more
10 mg/kg ACZ885	<20%	4	0	0
	20 to <50%	0	1	1
	50% or more	8	2	2
Placebo	<20%	9	1	0
	20 – <50%	2	0	0
	50% or more	3	0	0

Relationship between C-reactive protein (CRP) reduction and American College of Rheumatology (ACR) response at day 43 for patients in the 10 mg/kg group and patients receiving placebo. Note that two subjects are excluded from the 10 mg/kg ACZ885 because of missing data.

The serum concentrations of ACZ885 were determined in each dose group. The maximal concentration rose in a dose-proportional manner and reached a mean peak exposure of 329 μg/ml in the 10 mg/kg group after the second infusion, which decreased to less than 10 μg/ml by day 113. The elimination half-life was similar across all dose levels and averaged 21.5 ± 4.9 days (mean ± standard deviation). Details of exposure and its relationship to pharmacodynamic activities of ACZ885 will be reported elsewhere.

### Safety and tolerability

During the study five serious adverse events leading to hospitalization were identified. Among them, two events in the 10 mg/kg dose group (fracture of the left trochanter major after a bicycle fall and one case of tracheobronchitis) and one in the 0.3 mg/kg group (potential recurrence of angina pectoris) were considered not to be related to the study medication. Two episodes identified in the 1 mg/kg dose group (one case of erysipelas and one case of pulmonary infection) were considered to be related to study medication. All patients with infectious events were treated with antibiotics and recovered fully within the expected time frame. None of the patients prematurely discontinued treatment because of adverse events. There were no infusion-related adverse events; in particular, no anaphylactic or anaphylactoid reactions were reported. Serum samples were tested for the presence of anti-ACZ885 antibodies at baseline, on days 43 and 71, and at the end of the study (day 113). No formation of anti-ACZ885 antibodies was detected at any time point during the study.

The overall adverse event profile was almost the same for active and placebo treatment. Headache was reported less frequently with ACZ885 treatment (28.9% versus 46.7% for placebo), and urinary tract infection was reported frequently often with ACZ885 (21% versus 6.7% for placebo). Overall, intravenous administration of ACZ885 up to 10 mg/kg given twice with 14 days between dosing was found to be well tolerated without evidence of immunogenicity.

## Discussion

ACZ885, a novel human monoclonal antibody directed against human IL-1β, was found to be highly active in inhibiting IL-1β mediated arthritis in a preclinical animal model. The low ED_50 _observed in this model of aggressive inflammation is remarkable, because the injected recombinant cells constitutively secrete human IL-1β without any physiological feedback control. These results prompted us to study ACZ885 in a dose-escalating, proof-of-concept study in patients with RA – the first of its kind in humans. The study's primary objective was to investigate safety and tolerability; therefore, it was not powered to investigate efficacy or to draw firm conclusions regarding superiority or inferiority to treatment with anakinra or TNF blockers in RA. The small sample size of 38 patients treated with various doses of ACZ885 and 15 patients on placebo does not allow any definitive conclusion about safety to be drawn. Whether an increase in infectious episodes under ACZ885 represents an early sign of the consequences of blocking IL-1 – similar to observations with anakinra [21] – needs confirmation, and careful monitoring in upcoming larger studies is required. Immunogenicity and infusion-associated events were not observed with two intravenous administrations.

The study provides evidence of a treatment effect by demonstrating that 50% of patients achieved an ACR20 response up to day 43 in the highest tested dose of 10 mg/kg intravenous, along with a robust reduction in serum CRP. No firm conclusion regarding any dose-response relation can be drawn, because of the small sample size (*n* = 6) of the cohorts treated with 0.3, 1, and 3 mg/kg ACZ885. The onset of action in those who responded was found to be rapid. The majority of these responders satisfied the EULAR criteria for moderate or good response or ACR20 response criteria within 3 weeks. Similar to experience with other biologics for the treatment of RA patients, there was a subset of patients who did not respond to treatment. On the other hand, some patients achieved an ACR50 or even ACR70 response within a short period of treatment. This observation reflects the common understanding that RA represents a heterogeneous disease and that, even by applying strict inclusion criteria, the studied disease in clinical trials is not homogenous. Therefore, a better understanding of the pathophysiology on an individual basis is required if we are to better predict the response to a certain treatment and hence avoid treatment failures. Consequently, there is a need to establish biomarkers that have high predictive value; recent progress has been made for TNF blockers [22,23].

In the current study, CRP reduction was found to be necessary for achievment of clinical response, as expected, but it was not sufficient to explain or even predict clinical response, because CRP reduction was also observed in the absence of clinical response. Therefore, CRP reduction during treatment does not serve as a predictive biomarker for clinical responses at the individual level. Furthermore, levels of serum IL-6, TNF-α and IL-1Ra levels (as measured by enzyme-linked immunosorbent assay) were within the normal range in these patients and exhibited no conclusive changes during the course of the study (data not shown). At baseline, total IL-1β levels in serum were at or below the detection limit of the assay (about 1 pg/ml) and showed no difference between responders and nonresponders, indicating that baseline serum levels of these cytokines are of poor predictive value to identify treatment responders.

Almost all rheumatologists prefer to administer a TNF blocker rather than anakinra as a first biologic agent to treat RA patients, concordant with a recent consensus statement, and national and international registries [3]. Treatment with recombinant IL-1Ra (anakinra) has been shown to be effective in RA [24], but its efficacy appears to be lower than that of TNF-α inhibitors [25] and its administration is frequently associated with injection-related adverse events. Whether anakinra's lower efficacy is due to an inferior biologic role of IL-1 as compared with TNF-α in the pathogenesis of RA or is simply due to the fact that anakinra cannot fully neutralize IL-1 is currently unclear. ACZ885 can completely neutralize IL-1β over a long period of time; it therefore offers an opportunity to study the relative biological role of IL-1β in the pathogenesis of RA. Further and larger studies will determine the efficacy of ACZ885 in treating signs and symptoms in patients with RA, and they may allow comparison with the efficacies of other biologic agents.

## Conclusion

ACZ885 was found to inhibit IL-1β mediated joint inflammation in preclinical models. It was well tolerated in this first proof-of-concept study in RA patients and was associated with a significant treatment effect in about 50% of patients. Further studies are warranted to confirm these initial results, to establish a safety profile, to identify those patients who will profit most from blocking IL-1β, and to better understand the biological role played by IL-1β in the pathogenesis of RA.

## Abbreviations

ACR = American College of Rheumatology; CRP = C-reactive protein; DAS28 = Disease Activity Score using 28 joint counts; ED_50 _= dose effective to achieve a mean of 50% improvement; EULAR = European League Against Rheumatism; h = human; IL = interleukin; IL-1Ra = IL-1 receptor antagonist; RA = rheumatoid arthritis; TNF = tumour necrosis factor.

## Competing interests

This study was sponsored by Novartis Pharma AG, Exploratory Development, Basel, Switzerland. CR, AMW, TW, GJMB and TJ hold Novartis stock options and shares. WvdB has received Novartis funds to study cytokines in arthritis models. HG is author on patents related to ACZ885. GB and RA have acted as consultants for Novartis. The authors declare that they have no further competing interests.

## Authors' contributions

RA, JS, SW, GB and PvR conducted the clinical study as investigators and contributed to the writing of the manuscript. LJ, WvdB and HG performed the preclinical studies and contributed to the writing of the manuscript. TW and CR planned and initiated the clinical study and reviewed the data, YB wrote the trial protocol, MDL was the lead monitor, GJMB the lead pharmacokineticist and AMW the lead statistician. TJ reviewed and interpreted the data, wrote the manuscript and is responsible for the exploratory clinical development programme of ACZ885.
